# Effects of Different *Bacillus subtilis* Supplementation Levels on Fecal Microbiota and Metabolites in Goats

**DOI:** 10.3390/microorganisms13122740

**Published:** 2025-11-30

**Authors:** Anmiao Chen, Hu Liu, Jiancheng Han, Donghong Zhu, Shiyang Huang, Mao Li, Xiaoyan Deng, Ke Wang, Qun Wu, Yuanting Yang, Weishi Peng, Meng Zeng, Wenji Wang, Xiaosong Zhang, Hanlin Zhou

**Affiliations:** 1 Zhanjiang Experimental Station, Chinese Academy of Tropical Agricultural Sciences, Zhanjiang 524013, China; cam1835287831@163.com (A.C.); liuh2018@lzu.edu.cn (H.L.); hanjiancheng810@163.com (J.H.); 18376025735@163.com (X.D.); lp-wangke@163.com (K.W.); wuqun.2006@163.com (Q.W.); ytyang10@163.com (Y.Y.); m17378095519@163.com (W.P.); zmeng0909@163.com (M.Z.); 2 Institute of Agricultural and Animal Husbandry Industry Development, College of Animal Science and Technology, Guangxi University, Nanning 530004, China; 3 Agricultural Products Processing Research Institute, Chinese Academy of Tropical Agricultural Sciences, Zhanjiang 524013, China; jpf0212@163.com; 4 Tropical Crops Genetic Resources Institute, Chinese Academy of Tropical Agricultural Sciences, Haikou 571101, China; limaohn@163.com; 5 Department of Animal and Veterinary Sciences, Aarhus University, DK 8830 Tjele, Denmark; wangwj@anivet.au.dk; 6 College of Veterinary Medicine, China Agricultural University, Beijing 100083, China; zhangxsgs@163.com

**Keywords:** *Bacillus subtilis*, Leizhou goat, fecal microorganisms, metabolomics

## Abstract

This study investigated the effects of *Bacillus subtilis* (*B. subtilis*) supplementation on microbiota and metabolites in the feces of Leizhou goats. Eight Leizhou goats were used in a replicated 4 × 4 Latin square design according to their gender (nanny goats and billy goats) with a 4 × 2 factorial arrangement of treatments that included four *B. subtilis* additive doses (control [0 g/d; NC, BC], low [2.5 g/d, NL, BL], medium [5 g/d, NM, BM], and high [7.5 g/d, NH, BH]) and 28 d periods (*n* = 4 per group), each consisting of 27 d adaption and 1 d sample collection. After collecting 32 fecal samples, 16S rRNA gene sequencing and LC-MS were performed to analyze microbial composition and metabolites, respectively. At the genus level, the relative abundance of *Rikenellaceae_RC9_gut_group* was significantly higher (*p* < 0.05) in the NM group than in the NC group. The relative abundance of *Treponema* sp. was significantly lower (*p* < 0.05) in the NM group than in the NC group. In billy goats, the relative abundances of *UCG-005* and *Rikenellaceae_RC9_gut_group* were significantly higher (*p* < 0.05) in the BH group than in the BC group. The relative abundance of *Treponema* sp. was significantly lower (*p* < 0.05) in the BL, BM, and BH groups than in the BC group. Furthermore, metabolomic analysis revealed that *B. subtilis* significantly altered the concentrations of glucose metabolism modulators (1-deoxynojirimycin, 1-DNJ) and certain bioactive peptides. Many amino acid metabolic pathways were also enriched. Correlation analysis demonstrated close connections between differential metabolites and the top 10 bacterial genera in fecal samples. These results provide new insights into the impact of *B. subtilis* on the microbial community and metabolic profile of the feces of Leizhou goats. In this experiment, the appropriate doses of *B. subtilis* for nanny goats and billy goats were 5 g/d and 7.5 g/d, respectively, but the optimal doses still need to be verified based on performance-based feeding tests in the next study.

## 1. Introduction

Gut microbial diversity and richness serve as critical biomarkers reflecting host intestinal nutrient metabolism and immune function [1]. Historically, antibiotics have been widely used to promote growth and treat diseases in farm animals, yet their drawbacks have become increasingly apparent. First, antibiotics indiscriminately disrupt gastrointestinal microbes, compromising microbial balance and weakening gut homeostasis [2]. Second, residual antibiotics in wastewater or manure accumulate persistently, polluting aquatic ecosystems and soil if released untreated [3]. Moreover, antibiotic residues may persist in meat, eggs, and dairy products, posing a latent health risk to consumers [4]. Probiotics, frequently referenced in food science, constitute a diverse category of beneficial microorganisms for humans and animals, encompassing commonly utilized strains such as *lactobacilli*, *bacilli*, and *saccharomyces* [5]. Contemporary research confirms their multifaceted mechanisms for suppressing enteric pathogens, establishing them as the most efficacious antibiotic alternative for maintaining gastrointestinal health [6,7]. *B. subtilis* is a biologically active probiotic that is directly viable for animal feeding and is widely present in the gastrointestinal tract [8]. *B. subtilis* secretes digestive enzymes, such as cellulase and protease, which facilitate the breakdown of feed components, thereby enhancing nutrient digestibility and reducing energy loss in animals [9,10]. Previous studies have shown that *B. subtilis* can affect lipid metabolism by regulating the ratio of Firmicutes to Bacteroides [11]. Zou et al. [12] found that adding *B. subtilis* to the feed can enhance the intestinal barrier function in poultry and reduce intestinal damage caused by Perfringens perfringens infection. The compound preparation of *B. subtilis*, Saccharomyces cerevisiae, and Enterococcus faecalis more effectively increases milk production in dairy goats and improves milk composition compared to using each strain alone [13]. These are quite likely related to *B. subtilis*-mediated modulatory effects on the regulation of the expression of related genes and intestinal flora composition.

The Leizhou goat is native to the Leizhou Peninsula, from which it derives its name. It is a unique goat breed in southern China. The Leizhou goat has unique heat tolerance and natural resistance to diseases, making it an ideal goat breed for cultivation in tropical and subtropical regions. Its meat is delicious and widely loved by consumers [14]. The Leizhou goat grows slowly, and the main farming method currently involves grazing combined with supplementary feeding. The efficiency of meat production is insufficient to meet market demand. In addition, the prices of major agricultural products such as soybeans, corn, and alfalfa in China have risen continuously. Therefore, more nutritional strategies need to be studied to enhance the efficiency of livestock farming. For instance, feed additives can be used to adjust the structure of the intestinal flora, thereby facilitating the digestion and absorption of nutrients by the animals.

Although a large number of studies have confirmed that *B. subtilis* plays a role in promoting gastrointestinal health and productivity in animals, its specific effects on the intestinal microbiota and metabolites have not been fully explored. There was still a research gap regarding the comprehensive impact of *B. subtilis* on the intestinal microbiota –metabolite interaction in Leizhou goats. No prior work has systematically assessed how *B. subtilis* supplementation alters the fecal microbiome and metabolome in this breed, nor whether responses differ between males and females. Therefore, a comprehensive study on the effects of adding *B. subtilis* on the fecal microbiota and metabolome of Leizhou goats is necessary. Thus, the primary objective of this study was to investigate the effects of *B. subtilis* on hindgut microbiota and metabolism using an integrated approach combining 16S rRNA gene sequencing and LC-MS metabolomics. We hypothesized that the addition of *B. subtilis* to the diet would alter the community structure of fecal microbiota at the genus level and intestinal metabolic activities in Leizhou goats, and the responses would vary between different genders. Meanwhile, in this experiment, different addition levels were set to seek the appropriate addition amounts of *B. subtilis* for female and male Leizhou goats. In future research, we will verify the application effects of *B. subtilis* at different doses on the production performance, feed digestion, and healthy immunity of Leizhou goats. This study lacks research on the production performance, nutrient digestion, and healthy immunity of Leizhou goats. Further research is still needed.

## 2. Materials and Methods

### 2.1. Animals, Experimental Design, and Diets

The experiment was conducted at a goat farm, located in Zhanjiang, Guangdong Province, China, owned by the Zhanjiang Experimental Station (ZES), Chinese Academy of Tropical Agricultural Sciences. It began in July 2024 and lasted for 112 days.

Four Leizhou billy goats and four Leizhou nanny goats were selected from the herd. Body weight (BW) at the start of the experiment was 14.01 ± 0.61 kg (mean ± SEM) and 10.71 ± 0.62 kg for billy and nanny goats, respectively. A replicated 4 × 4 Latin square design based on gender was employed for this trial, with 4 periods; 28 days each (27 days adaptation, 1 day for sample collection). *B. subtilis* PB6 probiotic (1 × 10^13^ CFU/g) was provided by Kemin (China) Technologies Co., Ltd. (Zhuhai, China). According to the manufacturer’s instructions, four supplementary gradients were set, as follows: 0 times, 0.5 times, 1 times, and 1.5 times the recommended dosage. Female or male animals were randomly allocated to one of the four following dietary treatments: diet + 0 g/d *B. subtilis* (Control), diet + 2.5 g/d *B. subtilis* (Low), diet + 5 g/d *B. subtilis* (Medium), and diet + 7.5 g/d *B. subtilis* (High). This resulted in eight treatment combinations, as follows: NC, NL, NM, NH for nanny goats and BC, BL, BM, BH for billy goats (*n* = 4 per group).

The goats were fed the experimental diets twice daily at 8:00 a.m. and 4:00 p.m. The animals had free access to drinking water. The amount offered to the goats fed ad libitum was adjusted to maintain orts at 10% of the total offered. All goats were fed a basal diet of oat hulls, corn, and soybean meal-based pelleted total mixed rations (PTMRs), as shown in Table 1.

### 2.2. Sample Collection and Measurement

#### 2.2.1. Fecal Sample Collection

Fresh fecal samples were collected before the morning feeding by rectal palpation and were immediately transferred into sterile 10 mL centrifuge tubes and rapidly immersed in liquid nitrogen (−196 °C). Thereafter, they were stored in a −80 °C freezer for future microbiome and metabolome analyses.

#### 2.2.2. 16S rDNA Gene Sequencing Analysis

DNA extraction of 32 fecal samples was carried out using the E.Z.N.A.^®^ stool DNA Kit (Omega Bio-tek, Norcross, GA, USA) following the manufacturer’s instructions. The DNA extract was examined on 1% agarose gel, and DNA concentration and purity were determined by a NanoDrop2000 spectrophotometer (Thermo Scientific, Waltham, MA, USA). Primer pairs 338F (5′-ACTCCTACGGGAGGCAGCAG-3′) and 806R (5′-GGACTACHVGGGTWTCTAAT-3′) were used to amplify the V3-V4 region of the 16S rDNA gene using an ABI GeneAmp^®^ 9700 thermocycler PCR system (Applied Biosystems, Waltham, MA, USA). The PCR reaction mixture was prepared using 10 μL 2 × Pro Taq, 0.8 μL of each primer (5 μM), 10 ng template DNA, and ddH_2_O to a final volume of 20 μL. The PCR amplification cycle was performed as follows: initial denaturation at 94 °C (5 min), 27 cycles of denaturation at 94 °C (30 s), annealing at 55 °C (30 s), extension at 72 °C (45 s), a single extension at 72 °C (10 min), and end at 4 °C. The PCR product was extracted from 2% agarose gel, purified using the PCR Clean-Up Kit (YuHua, Shanghai, China), and quantified using Qubit 4.0 (Thermo Fisher Scientific, USA) according to the manufacturer’s instructions. Aliquots of the samples were pooled, purified, and sequenced on the Illumina Nextseq2000 platform (Illumina, San Diego, CA, USA) using PE250 read lengths. We clustered purified sequence data via UPARSE, established operational taxonomic units (OTUs) at 97% sequence identity, and annotated species through reference database comparisons using the RDP classifier algorithm. To reduce the interference of sequencing depth, we normalized microbial community profiles to a uniform depth, setting the sequence count threshold to the lowest observed sequence count across samples, and then assessed species richness and diversity.

#### 2.2.3. Analysis of Non-Target Metabolomics in the Feces

A 50 mg fecal sample, a 6 mm diameter grinding bead, and 0.4 mL of extraction solution (methanol:water = 4:1 (v:v)) containing 0.02 mg/mL of internal standard (L-2-chlorophenylalanine) were mixed in a 2 mL centrifuge tube. Samples were ground for 6 min at −10 °C, 50 Hz, followed by ultrasonic extraction for 30 min at 5 °C. The samples were stored at −20 °C for 30 min, centrifuged for 15 min (4 °C, 13,000× *g*), and the supernatant was transferred to the injection vial for LC-MS/MS analysis. In order to monitor the stability of the analysis, a quality control (QC) sample prepared by mixing all the samples in equal volumes was inserted every 15 samples.

Fecal samples were analyzed by LC-MS/MS using a Thermo UHPLC-Q Exactive HF-X system at Majorbio Bio-Pharm Technology Co., Ltd. (Shanghai, China). The mass spectrometric data were collected using a Thermo UHPLC-Q Exactive HF-X Mass Spectrometer equipped with an electrospray ionization (ESI) source operating in positive and negative modes. Data acquisition was performed in data-dependent acquisition (DDA) mode. Detection was carried out over a mass range of 70–1050 *m*/*z*.

LC/MS raw data were preprocessed using Progenesis QI (Waters Corporation, Milford, MA, USA) software (https://www.waters.com/nextgen/us/en/products/informatics-and-software/mass-spectrometry-software/progenesis-qi-software/progenesis-qi.html?srsltid=AfmBOoqeju1SLZTH5q7tQyE9x-M4POAk9Y8iccvWH-bmedE86riESk7A, accessed on 28 November 2025). Internal standard peaks, as well as any known false positive peaks (including noise, column bleed, and derivatized reagent peaks), were removed from the data matrix, and the data were then de-redunded and peak-pooled. At the same time, the metabolites were identified by searching the databases. The main databases were HMDB (http://www.hmdb.ca/, accessed on 30 August 2025), METLIN (https://metlin.scripps.edu/, accessed on 15 August 2025), and the Majorbio Database.

Metabolite features detected in ≥80% of samples were retained. Data were normalized by sum normalization, log10-transformed, and batch-corrected after removal of QC samples with RSD > 30%. OPLS-DA (R package “ropls”, version 1.6.2) was used to identify differential metabolites (VIP > 1, *p* < 0.05, unadjusted) using 7-fold cross-validation.

After quality control and preprocessing of the metabolomic raw data, differential metabolites were annotated in KEGG, screened, identified, and subjected to KEGG pathway enrichment analysis.

### 2.3. Data Analysis

Data were statistically analyzed using two-way analysis of variance in SPSS 25.0 software (IBM Corporation, Armonk, NY, USA). The statistical model was as follows:

$$Y_{ijkl}=\mu +D_{i}+G_{j}+{P_{l}+DG}_{ij}+e_{ijl}$$

where *Y_ijkl_* = observation; *µ* = overall mean for each parameter; *D_i_* = effect of dose; *G_j_* = effect of host gender; *P_l_* = effect of experimental period; *DG_ij_* = interaction between gender and dose; and *e_ijl_* = random error, used to test dose, host gender, and dose × host gender interactions. Spearman’s rank correlation was used to analyze the correlation between fecal microbiota and metabolites. Statistical differences were considered at *p* < 0.05.

## 3. Results

### 3.1. Microbial Community Structure of Feces

**Figure 1:**
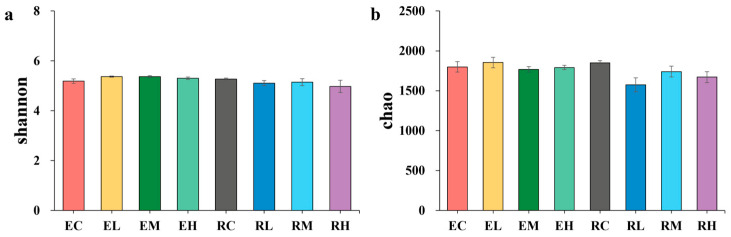
Shannon index box plot (**a**). Chao 1 index box plot (**b**). NC, NL, NM, and NH (nanny goats with 0, 2.5, 5, and 7.5 g *B. subtilis* in the diet, respectively); BC, BL, BM, and BH (billy goats with 0, 2.5, 5, and 7.5 g *B. subtilis* in the diet, respectively). (*n* = 4 per group).

A total of 2,655,495 raw sequences were detected in the fecal microbiota. After quality control, an average of 2,597,696 optimized sequences were obtained based on a 97% similarity threshold, yielding 5707 OTUs. The intersection number of OTUs (Figure S1a) among the eight groups was 1272, accounting for 22.29% of the total OTUs; the numbers of unique OTUs in the NC, NL, NM, NH, BC, BL, BM, and BH groups were 301, 367, 289, 247, 312, 134, 241, and 232, respectively. The rarefaction curve (Figure S1b) demonstrates a plateau, showing that the sequencing depth was sufficient to cover microbial diversity present in the 32 samples. The alpha diversity indices (Shannon index (Figure 1a) and Chao1 index (Figure 1b)) showed no significant differences among groups (*p* > 0.05), indicating that *B. subtilis* supplementation did not affect the richness and diversity of the fecal microbiota. PCA (principal component analysis) revealed that microbial community structure did not differ significantly between the control and treatment groups.

**Figure 2:**
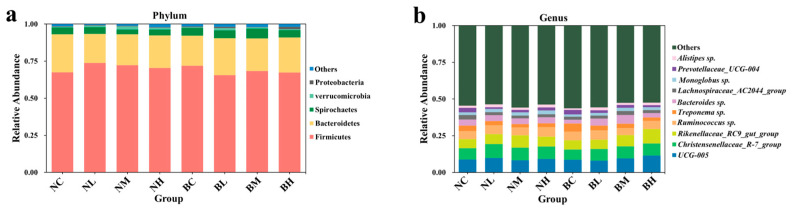
Microbial community structure in the feces of Leizhou goats on the phylum level (**a**) and genus level (**b**). NC, NL, NM, and NH (nanny goats with 0, 2.5, 5, and 7.5 g *B. subtilis* in the diet, respectively); BC, BL, BM, and BH (billy goats with 0, 2.5, 5, and 7.5 g *B. subtilis* in the diet, respectively). (*n* = 4 per group).

At the phylum level (Figure 2a, Table S3), Firmicutes (69.65%), Bacteroidetes (22.38%), Spirochaetota (4.77%), and Verrucomicrobia (1.04%) emerged as the dominant bacteria. At the genus level (Figure 2b, Table S3), the relative abundance exceeded 5% for *UCG-005* (9.23%), *Christensenellaceae_R-7_group* (8.25%), *Rikenellaceae_RC9_gut_group* (7.26%), and *Ruminococcus* sp. (5.70%) across the eight groups. Nanny goats in the NM group exhibited a significantly higher relative abundance of *Rikenellaceae_RC9_gut_group* compared to the NC group (*p* < 0.05), while *Treponema* sp. showed a significantly lower relative abundance under the same conditions (*p* < 0.05). In billy goats, the relative abundance of *UCG-005* and *Rikenellaceae_RC9_gut_group* increased significantly in the BH group compared to the BC group (*p* < 0.05). Relative to the BC group, *Treponema* sp. was markedly reduced in BL, BM, and BH groups (*p* < 0.05). The relative abundance of *UCG-005*, *Rikenellaceae_RC9_gut_group*, and *Treponema* sp. was significantly influenced by the interaction between the dose of *B. subtilis* and the gender of Leizhou goats (*p* < 0.05).

### 3.2. Fecal Metabolomics

**Figure 3:**
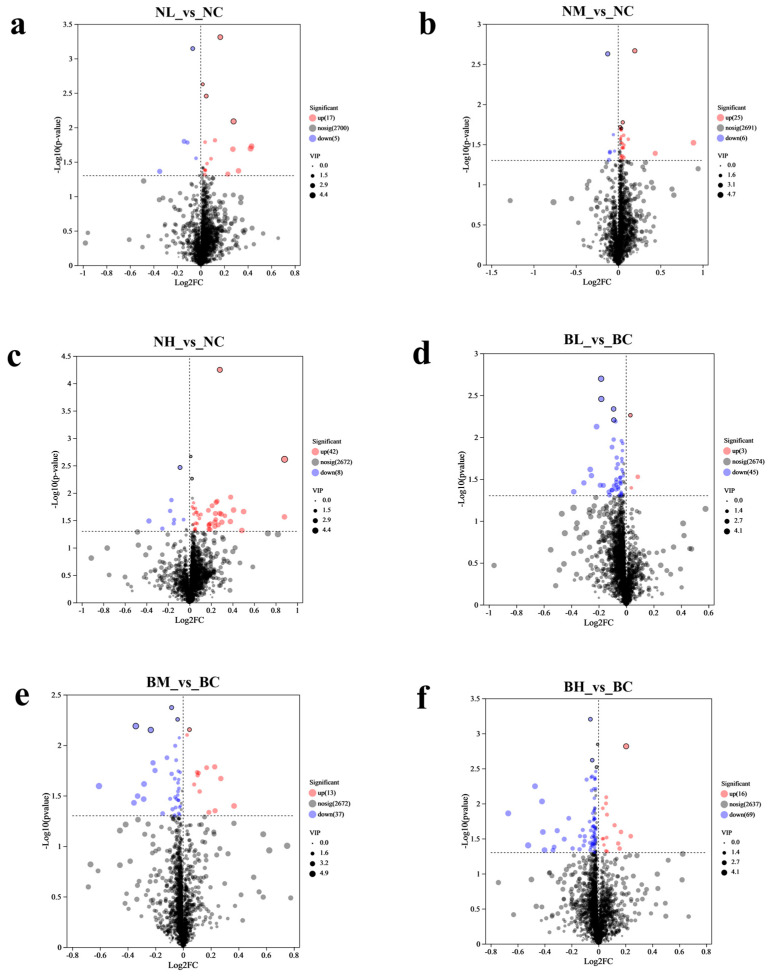
Volcano plots showing significantly differentially expressed metabolites for NC vs. NL (**a**), NC vs. NM (**b**), NC vs. NH (**c**), BC vs. BL (**d**), BC vs. BM (**e**), and BC vs. BH (**f**). NC, NL, NM, and NH (nanny goats with 0, 2.5, 5, and 7.5 g *B. subtilis* in the diet, respectively); BC, BL, BM, and BH (billy goats with 0, 2.5, 5, and 7.5 g *B. subtilis* in the diet, respectively). (*n* = 4 per group).

A total of 2722 metabolites were identified across the 32 fecal samples, with 1420 detected in the positive ion state (pos) while 1302 were identified in the negative ion (neg) state. Based on these metabolites, 20 third-level metabolic pathways were enriched, with the majority belonging to the “Metabolism” category (Figure S4). Variables with VIP > 1 and *p*-value < 0.05 were selected as potential metabolites. Relative to the NC group, the NL group (Figure 3a and Figure S5a) exhibited upregulated levels of 17 metabolites (including Trp-Lys-Tyr-Met-Val-D-Met, lyciumin B, and bis-ferulamidobutane), while 5 metabolites (such as 1-(3-methoxy-2-nitrostyryl) pyrrolidine, ferrostatin-1, and oxytetracycline) were significantly downregulated. In contrast to the NC group, the NM group (Figure 3b and Figure S5b) demonstrated increased levels of 25 metabolites (notably, 1-DNJ, oxaprozin, and Cyclo(le-Ala)), whereas 6 metabolites (particularly ferrostatin-1, Val Val Thr, and Dg (Pgf2Alpha/0:0/-22:0)) were markedly reduced. When compared with the NC group, the NH group (Figure 3c and Figure S5c) displayed increased levels of 42 metabolites (including 2-Galloyl-1,4-galactarolactone methyl ester, oxaprozin, and 1-isopropyl-1,2,3,4, -tetrahydro-beta-carboline-3-carboxylic acid); conversely, 8 metabolites were depressed. Differing from the BC group, the BL group (Figure 3d and Figure S5d) presented increased levels of 3 metabolites (including 1-DNJ and oxytetracycline), whereas 45 metabolites (specifically fumonisin B2, uric acid, and Cer (8:1_20/24:4)) were downregulated. The BC group served as the baseline; the BM group (Figure 3e and Figure S5e) showed increased levels of 13 metabolites (oxytetracycline, 3-(2,3-dihydroxypropyl)-6,8-dihydroxyisochromen-1-one, and Val Val Thr), but 37 metabolites (Glu-Lys-His, andrographolide, and enoxacin) displayed diminished concentrations. Unlike BC, the BH group (Figure 3f and Figure S5f) showed increased levels of 16 metabolites (Val Val Thr and 1-lsopropy1-1,2,3,4, -tetrahydro-beta-carboline-3-carboxylic acid), despite a simultaneous reduction in 69 metabolites (Glu-Lys-His, (1R,11S)-10-(thiophene-3-carbonyl)-3,10-diazabicyclo [9.3.0] tetradecan-2-One, and fumonisin B2).

**Figure 4:**
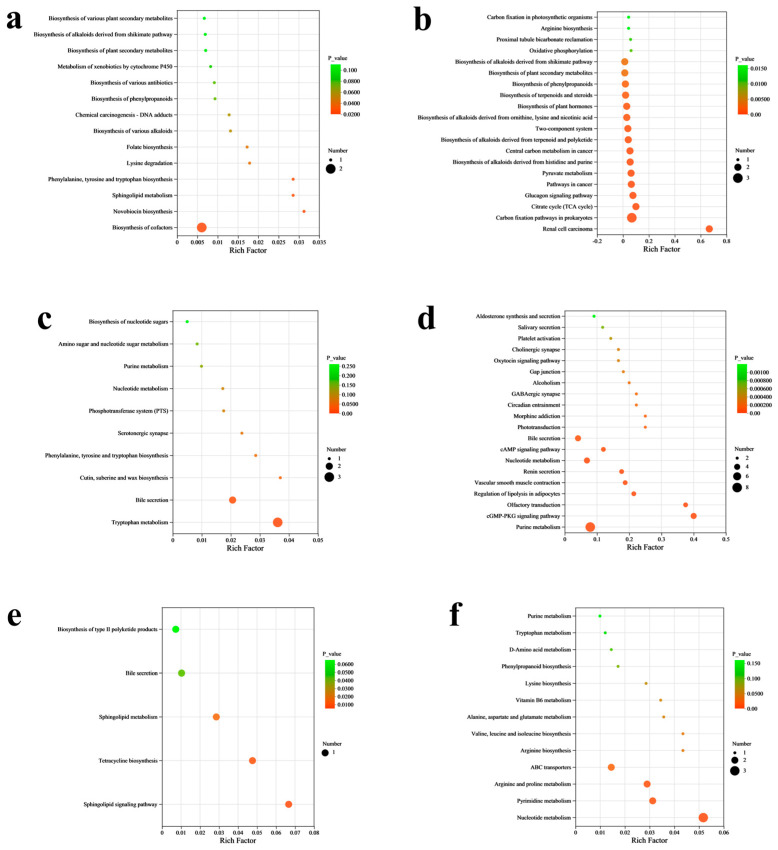
KEGG pathway enrichment analysis of differentially expressed metabolites for NC vs. NL (**a**), NC vs. NM (**b**), NC vs. NH (**c**), BC vs. BL (**d**), BC vs. BM (**e**), and BC vs. BH (**f**). NC, NL, NM, and NH (nanny goats with 0, 2.5, 5, and 7.5 g *B. subtilis* in the diet, respectively); BC, BL, BM, and BH (billy goats with 0, 2.5, 5, and 7.5 g *B. subtilis* in the diet, respectively). (*n* = 4 per group).

We performed KEGG pathway enrichment analysis of the differential metabolites and visualized the results using bubble plots. Lysine degradation emerged as the most significantly enriched pathway between NL vs. NC groups, followed sequentially by phenylalanine, tyrosine and tryptophan biosynthesis, sphingolipid metabolism, novobiocin biosynthesis, and biosynthesis of cofactors (Figure 4a). In the NM vs. NC groups, the glucagon signaling pathway, citrate cycle (TCA cycle), and carbon fixation pathways in prokaryotes were the significantly enriched metabolic pathways (Figure 4b). The main metabolic pathways altered between the NH and NC groups were serotonergic synapse; phenylalanine, tyrosine, and tryptophan biosynthesis; cutin, suberin, and wax biosynthesis; bile secretion; and tryptophan metabolism (Figure 4c). Comparative analysis between the BL and BC groups revealed significant perturbations in multiple metabolic pathways, including vascular smooth muscle contraction, regulation of lipolysis in adipocytes, olfactory transduction, cGMP-PKG signaling pathway, and purine metabolism (Figure 4d). Differential metabolites between the BM and BC groups were enriched in several pathways, such as biosynthesis of type II polyketide products, bile secretion, sphingolipid metabolism, tetracycline biosynthesis, and sphingolipid signaling pathway (Figure 4e). Lastly, KEGG pathway enrichment analysis between the BH and BC groups revealed that differential metabolites were mainly enriched in the metabolic pathways of arginine biosynthesis, ABC transporters, arginine and proline metabolism, pyrimidine metabolism, and nucleotide metabolism (Figure 4f).

### 3.3. Analysis of Differential Metabolites and Fecal Microbial Correlations

**Figure 5:**
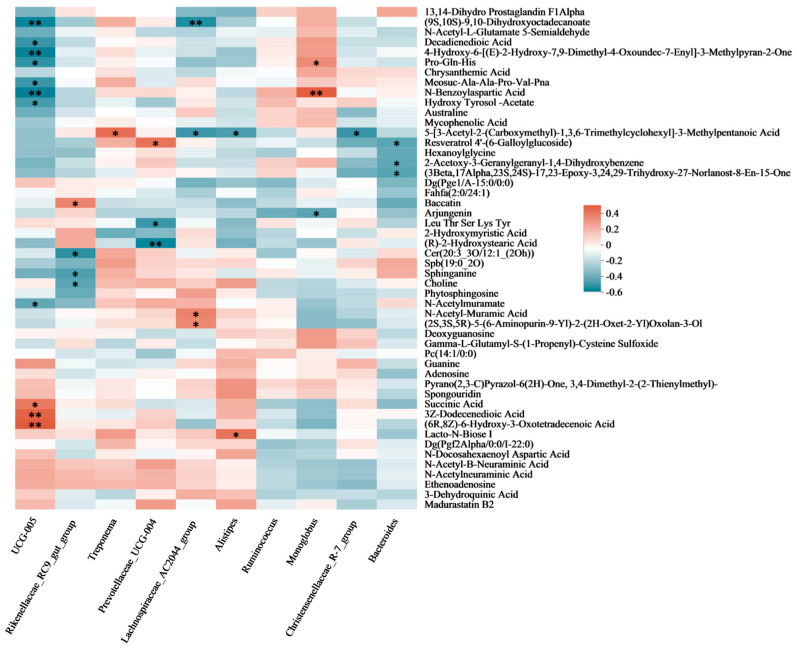
Correlation heatmap between differential metabolites and the top 10 microbial genera at the genus level. * and ** indicate significant differences at *p* < 0.05 and <0.01, respectively.

To further analyze the impact of *B. subtilis* on the intestinal tract, Spearman’s correlation analysis was conducted on the top 10 genera and differential metabolites. The results demonstrated the correlation between the microbiota and metabolites, further highlighting the obtained findings, especially the determination of the association between each genus and metabolite. As shown in Figure 5, *UCG-005* showed a significant negative correlation with eight different metabolites, such as (9S,10S)-9,10-dihydroxyoctadecanoate, decadienedioic acid, and 4-hydroxy-6-[(E)-2-hydroxy-7,9-dimethyl-4-oxoundec-7-enyl]-3-methylpyran-2-one. However, it was significantly positively correlated with three different metabolites, such as succinic acid and 3Z-dodecenedioic acid. *Rikenellaceae_RC9_gut_group* was positively correlated with baccatin, whereas it was significantly negatively correlated with three differential metabolites, such as Cer (20:3_3O/12:1_(2Oh)). *Treponema* sp. was positively correlated with mycophenolic acid.

## 4. Discussion

Gastrointestinal microorganisms are regarded as the main forces responsible for degrading nutrients in feed; they play a significant role in the nutritional metabolism, digestion, and absorption in ruminants [15]. Their composition is complex and diverse, and is influenced by gender, age, environment, and diet [16]. Our analysis revealed no significant differences in α-diversity among groups supplemented with varying doses of *B. subtilis*. In this experiment, Firmicutes and Bacteroides were the dominant phyla, and the sum of their relative abundance exceeded 90%, which is consistent with previous studies [17,18,19]. In the intestinal microbiota systems of mammals such as humans, rats, and pigs, Firmicutes and Bacteroides also hold a dominant position [20,21]. Unlike in mammals, Firmicutes and Bacteroides account for a high proportion of the intestines of poultry, and a large number of Proteobacteria are also present [22]. Previous evidence indicated that Firmicutes and Bacteroides phyla play significant roles in the intestinal ecosystem [23]. These two dominant bacterial phyla in the intestines play different roles in nutrient degradation and fermentation [24,25]. Firmicutes form the core fibrolytic bacterial consortium, which secretes cellulases and hemicellulases to break down recalcitrant polysaccharides into fermentable substrates (e.g., glucose and oligosaccharides), thereby enhancing dietary energy harvest [26]. More specifically, Firmicutes mediate lipid deposition through (1) regulation of energy metabolism, (2) promotion of short-chain fatty acid (SCFA) absorption, (3) modulation of bile acid metabolism, and (4) tuning of host inflammatory responses [27,28,29]. Concurrently, Bacteroidetes contribute to complex carbohydrate digestion and organic matter fermentation [30]. Published evidence demonstrates significant differences in Firmicutes-to-Bacteroidetes ratios between obese and lean mice (elevated in obesity), a pattern also observed in horse and human gut microbiomes [31,32,33]. In nanny goats, the relative abundance of Firmicutes was higher in the NL, NM, and NH groups, whereas that of Bacteroidetes was lower compared to the control group. Research shows that a higher F/B ratio is a sign of excessive fat deposition in the body [34]. However, the effect is different in billy goats. After adding *B. subtilis*, the F/B ratio in the treatment group decreased, especially in the BL group. This indicates that for nanny goats, the addition of *B. subtilis* may be beneficial for fat deposition. This might be due to the different sensitivities of the intestinal flora caused by gender differences [35]. It might also be a statistical deviation caused by the small number of experimental animals used in this trial.

Further analysis at the genus level showed that *UCG-005*, *Christensenellaceae_R-7_group*, and *Rikenellaceae_RC9_gut_group* were the dominant genera in fecal microbiota. The addition of *B. subtilis* significantly stimulated the proliferation of *UCG-005* and *Rikenellaceae_RC9_gut_group*, while suppressing the relative abundance of *Treponema* sp. Notably, *UCG-005* exhibited a positive correlation with animal growth performance and body weight metrics [36]. Diarrhea in lambs can cause a significant reduction in their relative abundance, leading to a decrease in feed efficiency [37]. As a recognized beneficial commensal, *UCG-005* demonstrates both anticoccidial properties and enteroprotective functions, likely mediated through competitive exclusion of opportunistic pathogens [38,39]. It is well known that *UCG-005* is a specialized bacterium that degrades and ferments complex polysaccharides to produce SCFAs [34]. SCFAs can be used by goats as an energy source to support growth, and they can also activate immune cells and regulate the expression of pro-inflammatory factors IL-6, IL-12, and TNF-α [40]. Parallel observations indicated that *Rikenellaceae_RC9_gut_group* modulates host inflammatory sensitivity—a phenomenon potentially attributed to *B. subtilis*-induced enhancement of secretory IgA (sIgA) production, thereby fortifying resistance against enteric pro-inflammatory bacteria [41,42,43]. The higher relative abundances of *UCG-005* and *Rikenellaceae_RC9_gut_group* suggest that these taxa contribute to improved host energy utilization efficiency and intestinal health. The relative abundance of *Treponema* sp. in billy goats fed with *B. subtilis* was significantly reduced. Previous studies suggested that *Treponema* sp. lysis elevates ruminal free lipopolysaccharide (LPS) concentrations, predisposing hosts to systemic inflammation [44]. This aligns with prior findings where dietary monensin and cashew nut shell extract (CNSE) similarly reduced *Treponema* spp. prevalence [45]. The appearance and evolution of antibiotic-resistant bacteria is one of the most serious problems associated with the use of antibiotics to kill or inhibit pathogenic bacteria; antibiotics can also disrupt the microbiota and impose a burden on the health of animals [46,47]. Research had indicated that probiotics were conducive to forming a synergistic or antagonistic effect with intestinal bacteria, further improving the intestinal environment and function [48,49,50]. Current evidence indicates that probiotics could potentially benefit animal growth performance and gastrointestinal function.

In our study, there was a significant interaction between the changes in the relative abundances of *UCG-005*, *Rikenellaceae_RC9_gut_group*, and *Treponema* sp. This indicates that the effect of *B. subtilis* is gender-specific. This observed difference may stem from sex-specific physiological mechanisms governing host responses to dietary *B. subtilis* supplementation. Gut microbiota diversity is significantly correlated with sex hormones and their metabolites [51,52]. Notably, the colonization dynamics of *B. subtilis* in the intestinal tract might be modulated by estrogens, progesterone, and androgens. Moreover, the typically higher feed intake and shorter intestinal retention time in rams may represent other limiting factors affecting the colonization efficiency and functional efficacy of *B. subtilis* in the gastrointestinal tract.

To elucidate the physiological impact of *B. subtilis* on gastrointestinal function, we conducted fecal metabolomic analyses. As terminal products of host–microbiota co-metabolism, fecal metabolites directly reflect feed efficiency, intestinal homeostasis, and systemic metabolic status [53]. Notably, both male and female goats exhibited elevated 1-DNJ levels following *B. subtilis* supplementation. Previous studies have shown that Bacillus can produce 1-DNJ—a potent inhibitor of α-amylase and α-glucosidase that delays glucose absorption, thereby exerting hypoglycemic effects [54,55,56]. *B. subtilis* may modulate glucose metabolism in Leizhou goats through a 1-DNJ-mediated mechanism. Furthermore, the experimental results showed that there are significant changes in the metabolic levels of various polypeptides, which play a significant role in interfering with the growth and development of the body, immune regulation, and metabolism. Certain peptides demonstrated antimicrobial, anticarcinogenic, and hypolipidemic properties [57,58]. These shifts likely stem from probiotic-induced modifications in protein digestion and microbial proteolysis, though mechanistic pathways warrant further investigation. Another interesting point is that supplementation with 2.5 g/d of *B. subtilis* markedly upregulated folate biosynthesis; folate is a nutrient whose demand increases during gestation. This enhanced microbial folate production could potentially support reproductive efficiency in does, though direct confirmation of breeding performance improvements would require further investigation. Finally, our research utilized a combined analysis of the microbiome and metabolism, and found that *UCG-005* has a significant correlation with succinic acid, an intermediate of the tricarboxylic acid cycle. This discovery reveals the possible pathways by which *B. subtilis* regulates the energy metabolism of the organism. However, the potential mechanisms by which *B. subtilis* regulates intestinal microbiota and metabolite composition and promotes the growth and health of Leizhou goats still require further research.

Although the combined analysis of the fecal microbiome and metabolome in this study provided basic data on the relationship between intestinal microbial diversity and structure and host metabolism in goats, the sample size of this experiment was relatively small, and it still needs to be verified in a larger population. Furthermore, due to the limitations of objective conditions, we were unable to conduct long-term follow-up investigations on the experimental animals, and the growth performance and health of the animals could not be measured. More data need to be measured and explored, such as rumen microbiota, rumen metabolism, blood metabolism, and host genomics, and the combined analysis of these datasets is required to explore multiple interaction pathways of *B. subtilis* in relation to the production performance and health of Leizhou goats.

## 5. Conclusions

The results presented in this article provide new insights into the impacts of *B. subtilis* on the fecal microbiota and metabolites. The addition of *B. subtilis* alters the composition of the bacterial community, increasing the relative abundance of *UCG-005* and *Rikenellaceae_RC9_gut_group*, while reducing the relative abundance of *Treponema* sp. Metabolomic analysis shows that the concentrations of 1-DNJ and peptide metabolites have significantly changed. Additionally, correlation analysis revealed potential links between bacterial genera and metabolites. These findings suggest potential effects on energy metabolism and intestinal health. Based on the microbiome and metabolome changes observed in this study, the doses producing the most substantial microbial and metabolic shifts were 5 g/d for nanny goats and 7.5 g/d for billy goats, but further performance trials are required before firm practical recommendations can be made.

## Figures and Tables

**Table 1:** Basal diet composition and nutritional level (air-dry basis/%).

Dietary Composition	%
Oat hulls	40.00
Corn	26.00
Soybean meal	12.40
Wheat bran	3.00
Soybean hulls	7.60
Palm meal	6.00
Soybean oil	1.00
Premix ^(1)^	4.00
Nutritional levels ^(2)^	
Metabolizable energy/ME (MJ/kg)	11.03
Dry matter, DM	95.91
Crude protein, CP	12.19
Ether extract, EE	6.63
Organic matter, OM	88.18
Neutral detergent fiber, NDF	51.97
Acid detergent fiber, ADF	20.12

^(1)^ The premix provided the following per kg of diet: VA 4 000 IU, VD3 1 600 IU, VE 36 mg, Cu 10 mg, Fe 50 mg, Zn 40 mg, Mn 42 mg, I 0.12 mg, Se 0.12 mg. ^(2)^ ME is a calculated value, while the others are measured values.

## Data Availability

The data used are confidential and will be made available upon request from the corresponding author (Hanlin Zhou).

## Supplementary Materials

The following supporting information can be downloaded at: https://www.mdpi.com/article/10.3390/microorganisms13122740/s1, Figure S1: Venn diagram of fecal bacterial OTUs (a). Rarefaction curve to indicate whether the amount of sequencing data in the sample is sufficient (b). Figure S2: Principal Component Analysis (PCA) analysis of nanny goats (a) and billy goats (b). Table S1: Microbial community alpha diversity. Table S2: The relative abundance (%) of fecal bacterial phylum of Leizhou goat. Table S3: The relative abundance (%) of fecal bacterial genus of Leizhou goat. Figure S3: OPLS-DA score plots and permutation tests results. Figure S4: KEGG pathway classification: metabolites detected and annotated. Figure S5: Variation important projection (VIP) of metabolites ranked according to metabolite importance in group differentiation. (a) NC vs NL, (b) NC vs NM, (c) NC vs NH, (d) BC vs BL, (e) BC vs BM, and (f) BC vs BH. NC, NL, NM, and NH (nanny goats with 0, 2.5, 5, and 7.5 g B. subtilis in the diet, respectively); BC, BL, BM, and BH (billy goats with 0, 2.5, 5, and 7.5 g B. subtilis in the diet, respectively). *n* = 4 per group.
