# Supplementary material for: Effects of Different Bacillus subtilis Supplementation Levels on Fecal Microbiota and Metabolites in Goats
Source: Microorganisms. 2025 Nov 30;13(12):2740. doi: 10.3390/microorganisms13122740 (PMC12736154; doi:10.3390/microorganisms13122740)
Supplement: Supplementary file 1 [file microorganisms-13-02740-s001.zip › microorganisms-3977234-supplementary.pdf]

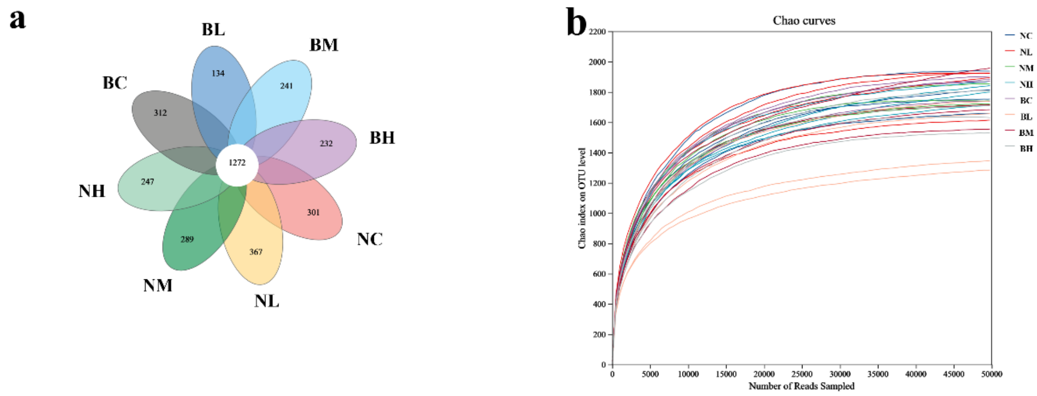

Figure S1: Venn diagram of fecal bacterial OTUs (a). Rarefaction curve to indicate whether the amount of sequencing data in the sample is sufficient (b). NC, NL, NM, and NH (nanny goats with 0, 2.5, 5, and 7.5g *B. subtilis* in the diet, respectively); BC, BL, BM, and BH (billy goats with 0, 2.5, 5, and 7.5g *B. subtilis* in the diet, respectively). n=4 per group.

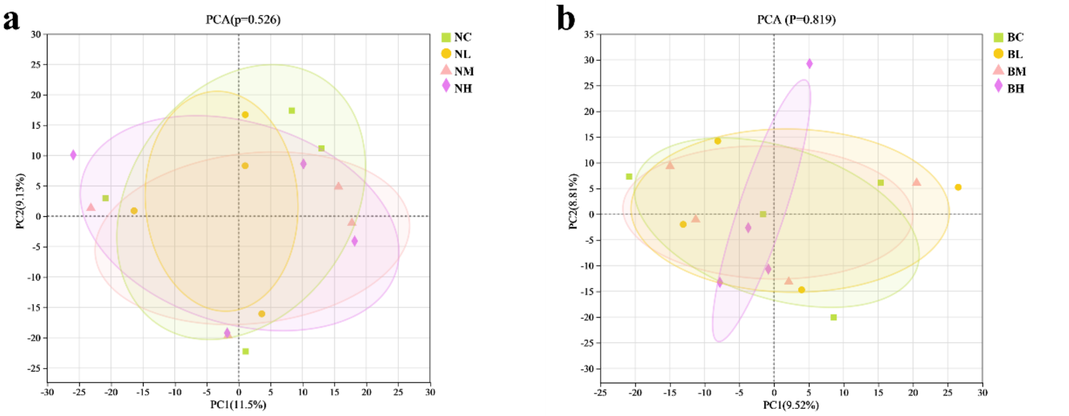

Figure S2: Principal Component Analysis (PCA) analysis of nanny goats (a) and billy goats (b). NC, NL, NM, and NH (nanny goats with 0, 2.5, 5, and 7.5g *B. subtilis* in the diet, respectively); BC, BL, BM, and BH (billy goats with 0, 2.5, 5, and 7.5g *B. subtilis* in the diet, respectively). n=4 per group.

Table S1: Microbial community alpha diversity. (n=4)

Item	Gender	Dose				SEM	*p*-value		
		Con	Low	Mid	High		Dose	Gender	Dose×Gender
Shannon	N	5.19	5.37	5.37	5.31	0.05	0.810	0.057	0.433
	B	5.26	5.14	5.10	4.98				
Chao l	N	1799.74	1768.92	1854.81	1792.03	21.38	0.187	0.069	0.058
	B	1849.45	1740.48	1574.95	1672.15				

N: nanny goat: B: billy goat; Con: diet + 0g/d *B. subtilis*; Low: diets + 2.5 g/d *B. subtilis*; Mid: diets + 5 g/d *B. subtilis*; High: diets + 7.5 g/d *B. subtilis*.

Table S2: The relative abundance (%) of fecal bacterial phylum of Leizhou goat. (n=4)

Item	Gender	Dose				SEM	*p*-value		
		Con	Low	Mid	High		Dose	Sex	Dose×Gender
Firmicutes	N	67.49	73.84	72.26	70.38	0.92	0.955	0.145	0.117
	B	71.94	65.62	68.30	67.35				
Bacteroidetes	N	25.66	19.59	20.83	22.01	0.69	0.830	0.594	0.062
	B	20.37	24.92	22.02	23.63				
Spirochaetes	N	4.48	4.57	3.24	3.93	0.47	0.983	0.166	0.763
	B	5.18	5.32	6.55	4.93				
verrucomicrobia	N	0.52	0.93	2.15	1.20	0.20	0.330	0.433	0.544
	B	0.46	1.41	0.98	0.66				
Proteobacteria	N	0.36	0.35	0.35	0.44	0.20	0.556	0.294	0.652
	B	0.47	0.91	0.20	1.69				
Others	N	1.48	0.72	1.17	2.04	0.17	0.642	0.220	0.451
	B	1.58	1.83	1.95	1.74				

N: nanny goat: B: billy goat; Con: diet + 0g/d *B. subtilis*; Low: diets + 2.5 g/d *B. subtilis*; Mid: diets + 5 g/d *B. subtilis*; High: diets + 7.5 g/d *B. subtilis*.

Table S3: The relative abundance (%) of fecal bacterial genus of Leizhou goat. (n=4)

Item	Gender	Dose				SEM	*p*-value		
		Con	Low	Mid	High		Dose	Sex	Dose×Gender
UCG-005	N	8.78	9.86	8.27	9.17	0.26	0.044	0.326	0.010
	B	8.62	8.02	9.62	11.50				
Christensenellaceae_R-7_group	N	7.72	9.53	8.64	8.55	0.23	0.154	0.109	0.744
	B	7.03	8.00	8.25	8.25				
Rikenellaceae_RC9_gut_group	N	6.21	6.73	8.42	6.70	0.27	0.001	0.197	0.003
	B	6.31	6.33	7.53	9.85				
Ruminococcus	N	5.51	5.98	5.27	6.41	0.23	0.380	0.696	0.735
	B	5.78	6.35	4.81	5.48				
Treponema	N	3.70	2.96	2.35	2.69	0.21	<0.001	0.019	0.025
	B	5.60	3.17	3.02	2.37				
Bacteroides	N	4.13	3.95	3.92	4.06	0.30	0.176	0.930	0.104
	B	2.53	4.79	5.93	3.00				
Lachnospiraceae_AC2044_group	N	3.01	2.06	1.94	2.12	0.21	0.881	0.905	0.622
	B	2.05	2.08	2.69	2.08				
Monoglobus	N	1.89	2.14	2.16	2.54	0.12	0.601	0.204	0.763
	B	1.65	1.85	2.16	1.83				
Prevotellaceae_UCG-004	N	3.06	1.19	1.72	2.08	0.22	0.063	0.888	0.757
	B	3.08	1.81	1.95	1.44				
Alistipes	N	1.42	2.03	1.51	1.87	0.19	0.599	0.680	0.988
	B	1.00	1.88	1.48	1.77				
Others	N	54.56	53.58	55.82	53.81	0.72	0.753	0.908	0.556
	B	56.36	55.72	52.55	52.42				

N: nanny goat: B: billy goat; Con: diet + 0g/d *B. subtilis*; Low: diets + 2.5 g/d *B. subtilis*; Mid: diets + 5 g/d *B. subtilis*; High: diets + 7.5 g/d *B. subtilis*.

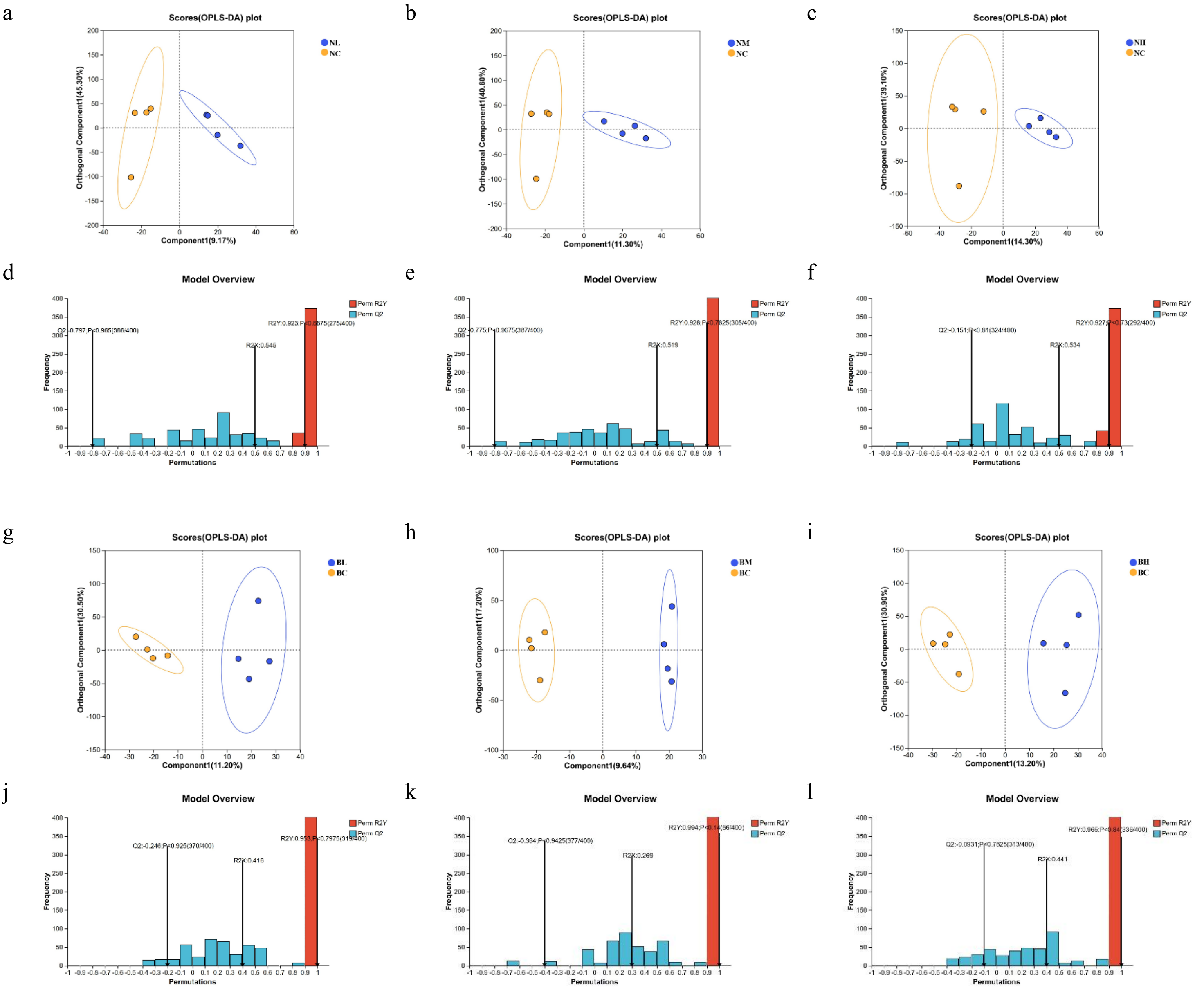

Figure S3: OPLS-DA score plots and permutation tests results. (a, d) NC vs NL, (b, e) NC vs NM, (c, f) NC vs NH, (g, j) BC vs BL, (h, k) BC vs BM, and (I, l) BC vs BH. NC, NL, NM, and NH (nanny goats with 0, 2.5, 5, and 7.5g *B. subtilis* in the diet, respectively); BC, BL, BM, and BH (billy goats with 0, 2.5, 5, and 7.5g *B. subtilis* in the diet, respectively). n=4 per group.

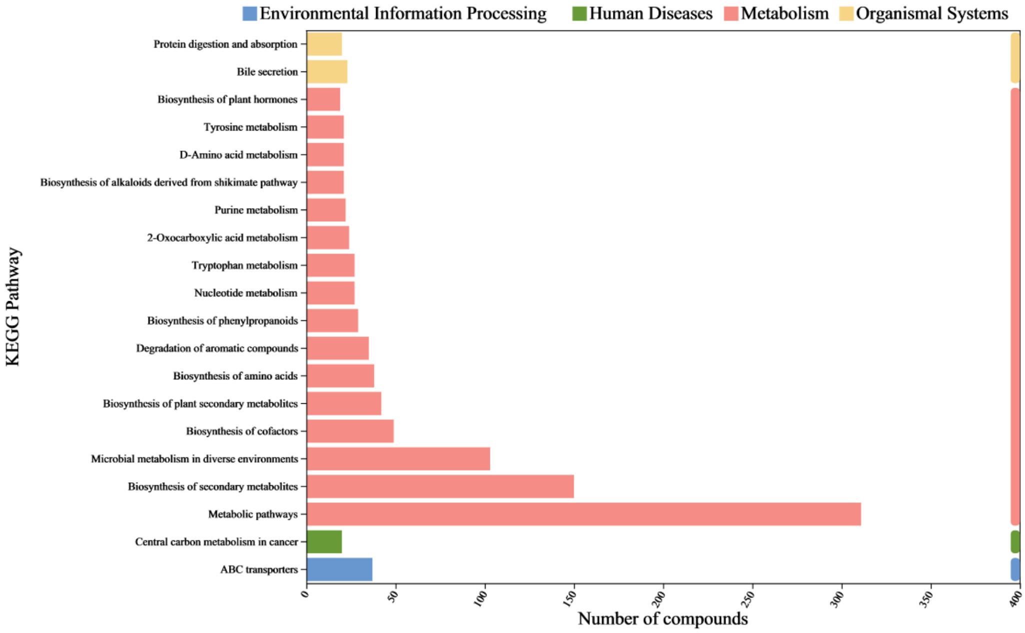

Figure S4: KEGG pathway classification: metabolites detected and annotated.

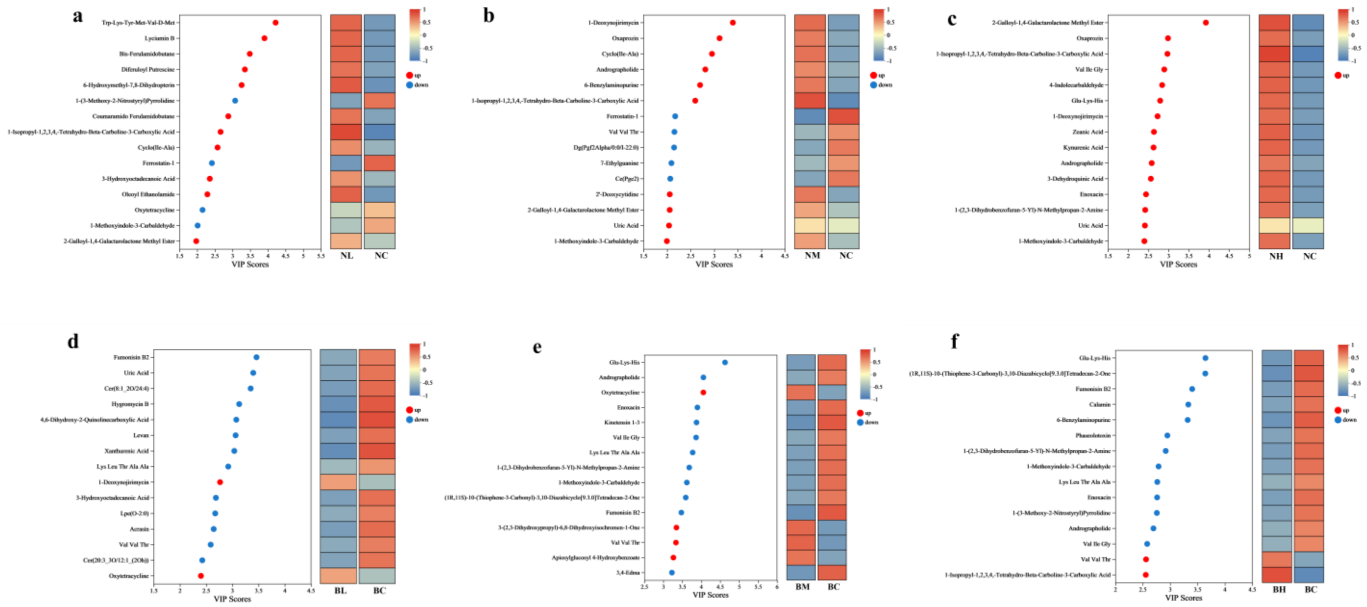

Figure S5: Variation important projection (VIP) of metabolites ranked according to metabolite importance in group differentiation. (a) NC vs NL, (b) NC vs NM, (c) NC vs NH, (d) BC vs BL, (e) BC vs BM, and (f) BC vs BH. NC, NL, NM, and NH (nanny goats with 0, 2.5, 5, and 7.5g *B. subtilis* in the diet, respectively); BC, BL, BM, and BH (billy goats with 0, 2.5, 5, and 7.5g *B. subtilis* in the diet, respectively). n=4 per group.
